# Silica Immobilised Chloro- and Amido-Derivatives of Eremomycine as Chiral Stationary Phases for the Enantioseparation of Amino Acids by Reversed-Phase Liquid Chromatography

**DOI:** 10.3390/molecules28010085

**Published:** 2022-12-22

**Authors:** Nikita Sarvin, Ruslan Puzankov, Georgii Vasiyarov, Pavel N. Nesterenko, Sergey M. Staroverov

**Affiliations:** 1 Department of Chemistry, M.V. Lomonosov Moscow State University, Leninskie Gory, 1, Moscow 119991, Russia; 2 CJSC BioChemMack S&T, Leninskie Gory, b.1/11, Moscow 119234, Russia

**Keywords:** glycopeptide antibiotic, chiral stationary phase, enantiomers, amino acids, eremomycin derivatives, high-performance liquid chromatography

## Abstract

Macrocyclic glycopeptide antibiotics immobilized on silica are one of the effective classes of stationary phases for chiral recognition and HPLC separation of a wide range of optically active compounds. Enantioselectivity primarily depends on the chemical structure of the chiral ligand, immobilization chemistry, and separation conditions. In the present work, three new chiral stationary phases (CSPs) based on macrocyclic antibiotic eremomycin were prepared and investigated for enantioseparation of amino acids. Two eremomycin derivatives, including simple non-substituted amide and bulky adamantyl amide, provided important information on the role of the carboxylic group in the eremomycin structure in the chiral recognition mechanism concerning amino acid optical isomers. One more CSP having a chlorine atom in the same position elucidates the role of the first aromatic ring in the eremomycin structure as a crucial point for chiral recognition. CSP with immobilized chloreremomycin was the most successful among the phases prepared in this work. It was additionally investigated under various separation conditions, including the type and content of the organic solvent in the eluent, the effects of different additives, and the concentration and pH of the buffer. Importantly, an efficient enantioselective separation of amino acids was achieved with pure water as the eluent.

## 1. Introduction

The vast majority of organic compounds associated with living organisms, as well as byproducts of their transformation and metabolism, are optically active. For example, natural amino acids, known as building blocks for peptides and proteins acting as enzymes, hormones, immunoglobilins, etc., can exist in two enantiomeric forms (D- and L-) having different biological activities. As a rule, L-amino acids dominate in living organisms, and the less abundant D-amino acids may have adverse effects [1]. In connection with the foregoing, determining enantiomeric purity is a significant task in biochemistry, biomedicine, pharmacology, and related areas.

Determination of the chiral configuration of these compounds is extremely important. High-performance liquid chromatography (HPLC) and capillary electrophoresis (CE) are commonly used techniques for the separation and determination of amino acid optical isomers. The chiral recognition of enantiomers required for their separation can be accomplished either in the mobile phase (CE or HPLC) or stationary phase (HPLC). Certainly, the latter option is more practical in terms of using small amounts of expensive chiral selectors immobilized on a surface for continuous use. The corresponding enantioselective adsorbents or chiral stationary phases (CSPs) can separate certain classes of compounds according to their ability to provide three-point interactions or chiral recognition [2]. No universal CSP allows the separation of all classes of organic compounds, so many sorbents with various immobilized chiral selectors have been proposed and investigated as CSPs [3]. The most popular CSPs have immobilized small ligands, such as polysaccharides [4,5], cyclodextrins [4,6,7], cinchona alkaloids [8,9,10], crown-ethers [11], Pirkle-type selectors [12,13], glycopeptide antibiotics [14,15,16], proteins [17,18], synthetic helical polymers [19], and various chiral frameworks [20].

The enantioselective separation of underivatized amino acids represents a difficult task due to the presence of rather acidic carboxylic groups and basic primary or secondary amino groups, which can dominate the retention mechanism and deteriorate three-point interactions in favor of a single-point interaction [21]. At present, this separation can be achieved by using CSPs containing immobilized zwitterionic cinchona alkaloids derivatives [9,22], crown ethers [10,23], polysaccharides [24,25], glycopeptide antibiotics [26,27], and amino acids in ligand exchange chromatography mode [28].

**Figure 1:**
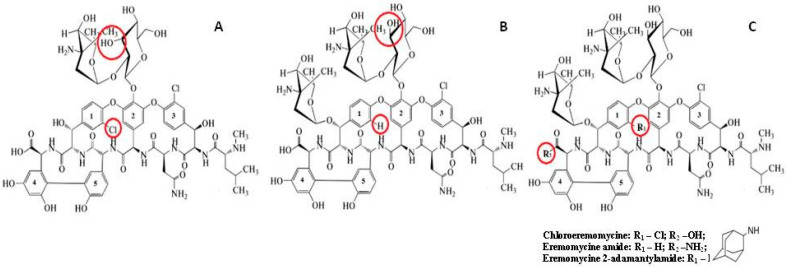
Chemical structures of vancomycin (**A**), eremomycin (**B**), and eremomycin derivatives used in this work (**C**). Structural differences are highlighted in red.

The family of silica-based CSPs with covalently attached macrocyclic or glycopeptides antibiotics (see their structures in Figure 1), including ristocetin A [27,29], eremomycin (E-CSP) [26,30], and teicoplanin (TE-CSP) [31], reveals a high selectivity for the separation of underivatized amino acid enantiomers. Chemical modification of the initial antibiotic molecule can drastically change the enantioselectivity. The modification of these antibiotics resulted in the preparation of CSPs with immobilized teicoplanin aglycone (TAG-CSP) [32] and eremomycin aglycone (EA-CSP) [29,30], which demonstrated different enantioselectivity for enantiomers of amino acids and β–blockers. For example, aromatic amino acids (Tyr, Phe, DOPA) enantiomers can be easily separated with E-CSP but not with EA-CSP [29].

The further methylation of TAG-CSP improved separation enantioselectivity for Phe and Trp derivatives having 5-hydroxy-, 5-methyl-, or 5-fluoro- substituent in the aromatic ring [33]. Several CSPs have been obtained by the chemical treatment of TE-CSP with phenyl- (Ph) [34], 3,5-dimethylphenyl- (DMP) [34], and *m*-tolyl- [35] isocyanates. It was suggested that these isocyanates reacted with hydroxyls in sugar units and with the amino- group in teicoplanin molecules and prepared phases, demonstrating increased hydrophobicity and the ability to form π-π interactions. The resulting enantioselectivity and resolution of amino acid enantiomers were better for the prepared CSPs as compared with TE-CSP, while DMP-TE-CSP was more selective than Ph-TE-CSP. The immobilization via hydroxyls makes available both amino- and carboxyl- groups from teicoplanin molecule for chiral recognition and hence the advantage of the zwitterionic nature of teicoplanin can be realized [36].

CSP with bonded vancomycin is the most useful for separating various derivatives of 2-phenylpropionic acid, also known as profens and amines, but not for underivatized amino acids [16]. There have been few attempts to improve the enantioselectivity of vancomycin-based phases by modifying their chemical structure. Novel EDP-V-CSP was prepared using vancomycin modified by Edman degradation to convert the secondary amino group having N-substituted leucine residue into the primary amino group [37]. The result was a noticeable increase in enantioselectivity compared to the native vancomycin containing CSP as reported for aromatic amino acids (DOPA, Phe). A similar effect was obtained for CSP with immobilized crystalline degradation products of vancomycin with additional carboxylic acid groups in the structure of the chiral selector [38]. It should be noted that the selectivity of CSPs with immobilized glycopeptides antibiotics depends strongly on the surface chemistry used for immobilization [39,40,41].

This study focuses on the investigation of three new CSPs with immobilized eremomycin derivatives. The main purpose of this work is associated with the identification of possible binding sites in eremomycin, which are responsible for the chiral recognition of underivatized amino acids. The influence of mobile phase composition on the enantioselectivity of the new CSP with chloreremomycin was also studied.

## 2. Results

### 2.1. Characterization of the Prepared CSPs

The chemical structure of eremomycin includes 22 chiral centers, 3 sugar fragments, 5 aromatic rings, 1 carboxylic group, 9 hydroxyl groups, 7 amide linkages, and 3 amino groups (Figure 1) [42]. The corresponding CSPs exhibit a high separation selectivity of amino acids enantiomers [15]. The chiral centers in the peptide core of the eremomycin molecule do not participate in the chiral recognition mechanism analogous with the structurally similar corresponding part of vancomycin, which does not demonstrate enantioselectivity to amino acids enantiomers [15]. The difference between eremomycin and vancomycin is associated with the presence of the additional chlorine atom in the first aromatic ring of the vancomycin molecule. Therefore, it was interesting to investigate the role of the chlorine atom in the same position as the eremomycin molecule in the chiral recognition of amino acids.

Another point to consider is connected with the possible effect of the carboxylic group location on the resulting enantioselectivity of the chiral selector. This acidic group can electrostatically interact with amino groups of amino acids. Synthetically, it is possible to locate the carboxylic group close to the additional sugar residue in the eremomycin molecule that allows elucidation of the role of the carboxylic group in combination with the sugar fragment in the chiral recognition of amino acids enantiomers.

**Figure 2:**
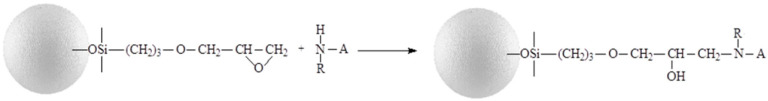
Immobilization of chiral selector (A) onto silica gel via the epoxy-group.

**Figure 3:**
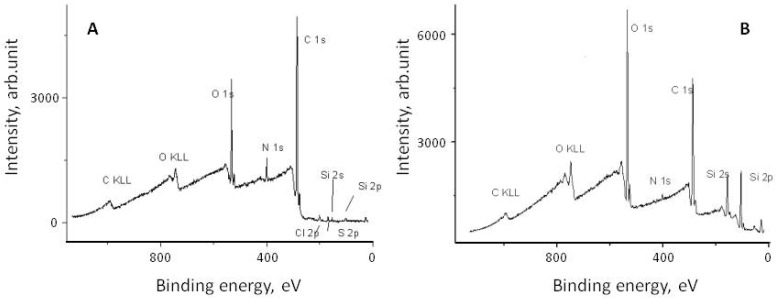
ESCA spectra of eremomycin (**A**) and corresponding stationary phase E-CSP (**B**).

It is believed that the attachment of eremomycin and its derivatives to the silica surface occurs via the reaction of the epoxy-group of activated silica and secondary amino-groups of glycopeptide antibiotics, as shown in Figure 2. Electron *spectroscopy* for chemical analysis (ESCA) was used to prove the covalent attachment of glycopeptide antibiotics to silica and the bonding chemistry of CSP. The corresponding spectra of eremomycin and E-CSP obtained with spectrometer XSAM800 (Kratos, UK) are presented in Figure 3. The increase in the relative intensity of Si2s, Si2p, and O1S peaks clearly indicates the presence of a silica matrix in E-CSP, while the presence of N 1s and C 1s peaks is associated with reduced concentration of eremomycin in the adsorbent.

The data of the elemental analysis of the prepared CSP are given in Table 1. The difference in carbon content (ΔC, %) between epoxy-activated silica and the resulting CSPs was used to calculate the surface concentration of eremomycin and its derivatives as described in [43]. The prepared epoxy-activated silica contained 7.1% C, and the resulting concentrations of the attached chiral selectors were in the range of 73.6–91.5 µmol/g.

A relatively long reaction time was applied at the final stage of CSP synthesis according to the strong requirement for carrying out the reaction under mild conditions to ensure the natural configuration of antibiotics is attached to the epoxy-activated silica surface. For this reason, the reaction occurred at room temperature for 4 days. The obtained values of surface concentrations of antibiotics of 0.18–0.22 groups per nm^2^ are close to the maximum possible coverage of the silica surface considering the size of antibiotic molecules [43]. Therefore the selected reaction time provided good yields for the attachment of antibiotics to epoxy-activated silica.

### 2.2. Comparison of Enantioselectivity for the Prepared CSPs

The immobilization of chiral selectors on epoxy-activated silica gel was performed under mild conditions from aqueous solutions with control of reaction yields and possible degradation products using RP-HPLC methods. This immobilization method allowed CSP preparation with a high surface concentration of chiral selectors.

The standard normal-phase HPLC procedure was used to evaluate the chromatographic performance of the prepared CSPs. The column efficiency was calculated from the chromatographic peaks of o-xylene eluted with 99:1 isooctane-isopropanol mixture with 28,030; 26,770; 26,890, and 28,160 theoretical plates per meter for E-CSP, eremomycin amide (Amide-E-CSP), (adamantyl-2) eremomycin amide (Adamantylmide-E-CSP), and chloreremomycin (Chloro-E-CSP), respectively.

As discussed in the Introduction, most macrocyclic glycopeptide antibiotic CSPs are enantioselective towards amino acids racemates. In this work, the enantioselectivity was tested for 22 amino acids, which were divided into four groups, including acidic (Asp, Glu), basic (His, Lys, Arg, Orn), aromatic (Trp, Tyr, Phe), and common acids (Ser, Thr, Cys, Met, Asn, Gln, Pro, Ala, Val, Leu, Ile, Nva, Nle). The concentration of amino acids was 0.1–0.2 mmol/L, depending on the response of the spectrophotometric detector. The retention and separation enantioselectivity of amino acids are discussed according to the classification mentioned above.

**Figure 4:**
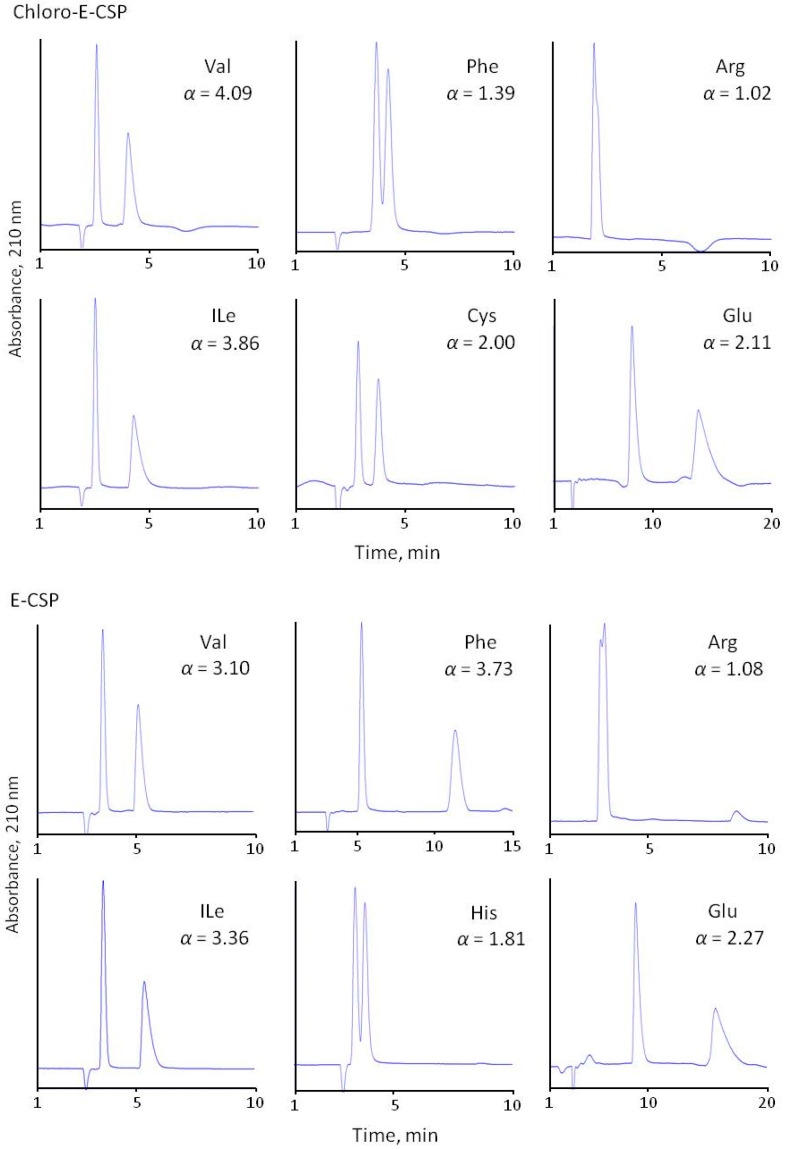

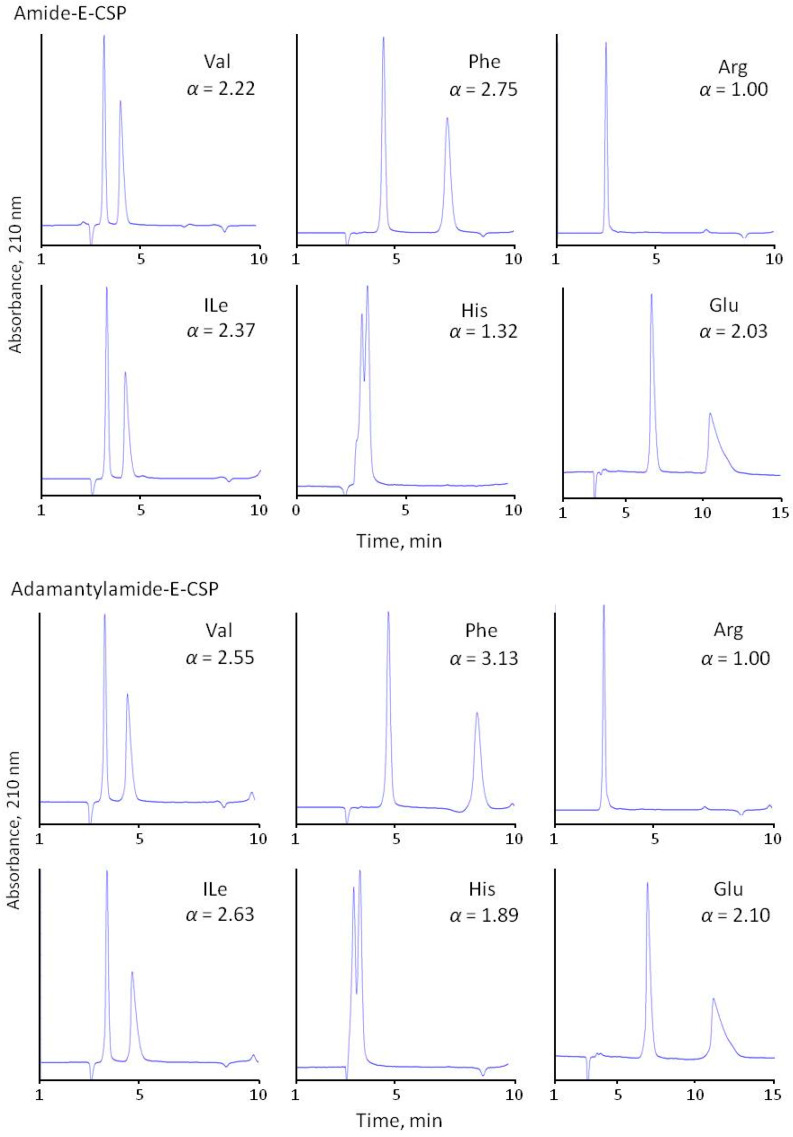
Separation of common (Val, Ile), aromatic (Phe, His), basic (Arg), and acidic (Glu) amino acids with four CSPs. Eluent 10% of methanol and 90% of 0.1 M sodium dihydrophosphate buffer with pH 4.5.

The separation selectivity of CSPs was evaluated compared to the chiral phase with immobilized eremomycin using 0.1 M of sodium dihydrophosphate buffer (pH 4.5) containing 20% methanol as the eluent. All prepared CSPs demonstrated high separation selectivity for the amino acid enantiomers (Figure 4 and Table 2). The obtained results are presented in Table 2. It should be noted that no retention was observed for basic diaminomonocarboxylic acids (Lys, Orn, Arg) having isoelectric points above 9.7, and only weak retention and separation were obtained for His (pI = 7.6) racemate. Clearly, this is due to the repulsion of amino acids positively charged at pH 4.5 from the positively charged eremomycin (isoelectric point circa 8.5) and its chloro- and amide derivatives bonding to silica. It should be noted that no reasonable resolution of basic amino acid enantiomers was obtained due to the poor retention of both enantiomers. A significantly reduced enantioselectivity was observed for aromatic amino acids with Chloro-E-CSP, indicating a small effect of π-π interactions in chiral recognition of amino acids enantiomers by CSPs. In truth, the aromatic amino acids Phe, Tyr, and Trp are less retained with Chloro-E-CSP than E-CSP (Table 2, Figure 4). The acidic amino acids are also retained stronger than common amino acids (see chromatograms of Glu in Figure 4). This is due to electrostatic interactions between negatively charged pH 4.5 Asp and Glu and positively charged surfaces of CSPs.

A significant decrease of 35–50% in retention times of amino acids was observed for amide-type CSPs, which can be explained by the conversion of polar carboxylic groups into neutral amide groups in the corresponding chiral selectors before immobilization. Obviously, the carboxylic group plays an important role in the retention of amino acids but has no apparent influence on the enantioselectivity of their separation (Table 2). This means that the combination of carboxylic groups with amino sugar residues does not play a significant role in the chiral recognition of amino acids.

Surprisingly, the adsorbent with immobilized chloreremomycin (Chloro-E-CSP) with a structure similar to vancomycin demonstrates better enantioselectivity compared with E-CSP for all of the amino acids, except aromatic amino acids (Trp, Tyr, Phe, His) and heterocyclic Pro (Figure 4 and Table 3). Enantioselectivity drops from 1.83, 4.30, and 3.79 to 1.10, 1.24, and 1.31 were noted for Trp, Tyr, and Phe with an 0.1 M phosphate buffer (pH 4.5) containing 10 *v*/*v*% methanol as eluent, respectively. Such a significant decrease in *α* value can be explained by the deterioration of π-π interactions between aromatic rings of amino acids and the aromatic ring in the first “basket” of the chiral selector. The presence of chlorine atoms in the aromatic ring of eremomycin can reduce this interaction due to the mesomeric effect. At the same time, the influence of the steric hindrance effect of rigid bulky aromatic and heterocyclic fragments in amino acids on chiral recognition cannot be ignored. A decrease in enantioselectivity from 9.0 to 2.7 for Pro and from 1.4 to 1.0 for His can be associated with steric hindrance. Importantly, enantioselectivity values for these amino acids on amide-type CSPs (Amide-E-CSP and Adamantylamide-E-CSP) are very close to those obtained with E-CSP.

### 2.3. Influence of Separation Conditions on Enantioselectivity of Chloro-E-CSP

The additional optimization of separation conditions was performed for Chloro-E-CSP as the most selective stationary phase among prepared CSPs. The parameters for both the aqueous (pH, type of additive, ionic strength) and the organic (content, solvent nature—methanol, ethanol, *n*-propanol, isopropanol, acetonitrile) components of the eluent were varied.

#### 2.3.1. Organic Solvent Type and Content

The effect of organic component type on enantioselectivity was investigated using mobile phases containing 10% organic solvents. It was found that the type of alcohol additive had practically no influence on enantioselectivity. The selectivity for aromatic amino acids slightly improved with changing from methanol to *n*-propanol. The use of acetonitrile resulted in a significant decrease in both enantioselectivity and peak resolution.

An increase in methanol concentration in the eluent from 0% to 70% caused a change in selectivity, with the maximum effect observed at 10–20% methanol content, depending on the amino acid type. For mobile phases with methanol content higher than 60%, the enantioselectivity decreased by more than two times. No separation was observed for aromatic amino acids (Trp, Tyr, Phe) with eluents containing more than 50% methanol.

Upon increasing the concentration of an organic component in the eluent, the retention times of amino acids increased, indicating the HILIC mechanism.

**Figure 5:**
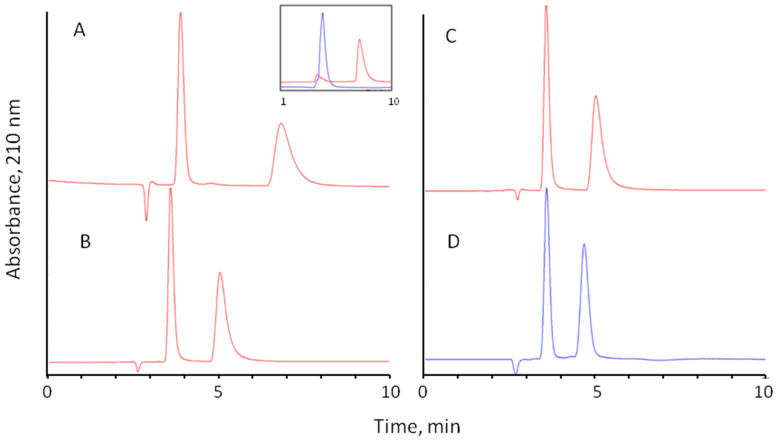
Separation of D,L-methionine on Chloro-E-CSP column with different mobile phases: (**A**)—0.1% perchloric acid (pH 4.5 adjusted with NH_4_OH)—methanol (90:10); (**B**)—water—methanol (90:10); (**C**)—water; (**D**)—0.1 M sodium phosphate (pH 4.5)—methanol (90:10). Column: 250 × 4.0 mm ID, flow rate 0.7 mL/min, temperature 25 °C; detection at 220 nm, injection volume 10 µL. Insert: separate chromatograms obtained for L-Met (blue chromatogram) and D-Met (red chromatogram) under the same conditions.

Interestingly, high enantioselectivity comparable with traditional buffers was observed for all investigated amino acids with pure water as the eluent (Table 3, Figure 5C). However, no elution was observed for acidic amino acids Asp and Glu in a reasonable time. Obviously, electrostatic interactions between negatively charged amino acids and positively charged surfaces of Chloro-E-CSP are responsible for strong retention in the eluent with no added electrolytes. Surprisingly, Asn, an amide of aspartic acid, was eluted at these conditions in a reasonable time (Table 3), confirming the crucial role of the second carboxylic group in the retention of Asp. It should be noted that the separation selectivity of Asp and Asn is similar to a 0.1 M phosphate buffer containing 10% methanol as the eluent. This means no contribution of the carboxylic or corresponding amide group in the chiral recognition mechanism.

In all experiments, the L-enantiomer of amino acid was eluted first, as confirmed by separate injections of pure enantiomers. The example of overlaid separations of methionine enantiomers is shown in the insert in Figure 5A.

**Figure 6:**
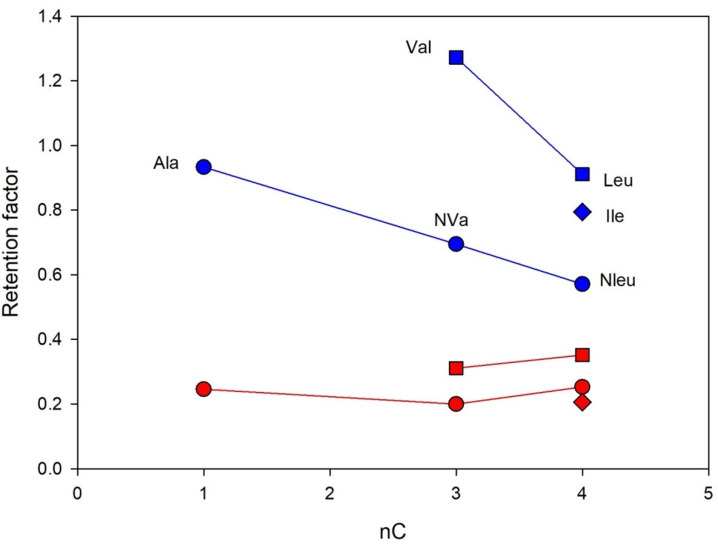
The retention of D- (blue lines) and L-enanatiomers (red lines) of amino acids containing alkyl groups in a side chain as a function of the number of carbon atoms. Column: Chloro-E-CSP, eluent 10% methanol and 90% of 0.1 M sodium dihydrophosphate buffer with pH 4.5.

The interesting effect of alkyl group structure in aliphatic amino acids (Ala, Val, Leu, Ile, Nle, NVa) on separation enantioselectivity was found. The effects of hydrophobic interactions and the size of the alkyl group expressed as a number of carbon atoms on the retention and separation enantioselectivity of Chloro-E-CSP were investigated for structurally similar amino acids with different alkyl- groups in the molecules, including methyl- (Ala), isopropyl (Val), *n*-propyl- (NVa), *n*-butyl (NLe), isobutyl (Leu), and *sec*-butyl- (Ile). The corresponding dependences obtained for *n*-alkyl- and *iso*-alkyl-containing amino acids are presented in Figure 6. Clearly, the increase in the alkyl group size decreases both retention and enantioselectivity.

#### 2.3.2. Effect of Different Additives

The eluent composed of 10% methanol and 90% of 0.1 M sodium phosphate buffer (pH 4.5) was used as a reference. The use of 0.1% triethylammonium (Et_3_N) with pH 4.5 adjusted by glacial acetic acid, 0.1% phosphoric acid, 0.1% trifluoroacetic acid (TFA), 0.1% acetic acid, and 0.1% perchloric acid with pH 4.5 adjusted by ammonia were tested as aqueous part of the eluents containing 10% methanol in all cases. The mobile phase based on perchloric acid demonstrated the best results with an increase of the enantioselectivity for aliphatic amino acids by an average of 20% compared with the reference eluent (Table 3, Figure 5D).

The other additives (trifluoroacetic, acetic and phosphoric acids, Et_3_N) tremendously reduced the enantioselectivity obtained with eluents containing 10% methanol. Among them, the buffer with trifluoroacetic acid demonstrated the best results (see Table 3).

#### 2.3.3. Buffer Concentration and pH Effects

The effects of pH and ionic strength on the enantioselectivity of Chloro-E-CSP were studied in the pH range from 3.6 to 8.4, with the concentration of phosphate buffer varied from 0.02 to 0.4 M. The best enantioselectivity for amino acids was obtained with a 0.2 M sodium phosphate buffer, except for aromatic amino acids where the optimum buffer concentration was 0.1 M. The increase of buffer concentration above 0.2 M resulted in the disappearance of enantioselectivity for Trp and Phe. The obtained values of enantioselectivity, retention factors, and resolutions are summarized in Table 4.

The phosphate buffer pH did not change the retention of amino acids except for acidic (Asp) and basic (His, Lys) amino acids. The retention factor of negatively charged Asp having two dissociated carboxylic groups decreased from 1.1 at pH 4.5 to 0 at pH 8.3 with methanol—0.1 M phosphate buffer (10:90). At the same time no retention of positively charged His and Lys was observed at pH 4.5, but both amino acids were weakly retained in the eluent with pH 8.3. Obviously, such behavior is associated with a decrease in the total positive charge of immobilized chloreremomycin due to the deprotonation of amino groups at high pH. This change in selector molecule charge resulted in decreased repulsion for positively charged His and Lys and increased repulsion for Asp. The reported pKa value of the amino group in the structurally similar vancomycin molecule was 7.78, supporting this explanation [44].

Basic amino acids His and Lys cannot be separated under standard conditions (0.1 M sodium phosphate buffer with pH 4.5, 10–20% methanol) on Chloro-E-CSP (Figure 4). However, their retention time began to grow along with the increasing pH of the buffer solution, and good separation selectivity of 1.4 and 1.5 was obtained at pH 8.3 (Table 4). For aromatic amino acids Trp and Phe, the best separation selectivity was obtained at pH 7.2. The enantiomers of other amino acids separated better with the reference mobile phase having pH 4.5.

## 3. Discussion

Three new CSPs containing immobilized eremomycin derivatives demonstrated enantioselectivity comparable to E-CSP. Overall for different types of amino acids, the best enantioselectivity was obtained with chloreremomycin containing CSP. Chloro-E-CSP revealed good enantioselectivity in different eluents, including the perchloric acid-based eluent, compatible with the MS detector. For the first time, amino acid enantiomers separation was achieved with pure water as the eluent.

The comparison of enantioselectivity obtained for CSPs with eremomycin and its derivatives allows the identification of key fragments in this selector, which are responsible for the chiral recognition of amino acids. Firstly, it can be concluded that the carboxylic group in E-CSP does not play an important role in the chiral recognition of amino acid enantiomers, while the first aromatic rings are actively involved in this process. Secondly, introducing a chlorine atom in the first aromatic ring (see Figure 1) significantly increases the enantioselectivity of CSP for all amino acids except for aromatic Trp, Phe, Tyr, His, and cyclic Pro. The substantial decrease in enantioselectivity for the latter group of amino acids was noted due to the presence of bulky rigid fragments in their molecules (Table 3 and Figure 4). Finally, the increase of the alkyl group size in a side chain of amino acids decreases both the retention and enantioselectivity of Chloro-E-CSP, as shown in Figure 6.

The investigation of pH and ionic strength effects showed that the optimum separation could be achieved near the isoelectric points (pIs) of acidic or alkaline amino acids. The separation of other amino acids enantiomers was better with buffers having pH above isoelectric points.

Recently, the enantioseparation of profens was studied with this set of CSPs and a very different chiral recognition mechanism was observed [45]. This study confirms a very complex enantioseparation mechanism for the prepared CSPs with different options for other class of organic compounds.

## 4. Materials and Methods

### 4.1. Materials and Chemicals

Chromatographically pure eremomycin amide and (adamantyl-2) eremomycin amide were provided by the Gause Institute of New Antibiotics (Moscow, Russia). Chloreremomycin was isolated from the cultural liquid provided by JSC «Biohimik» (Saransk, Russia) and purified using preparative reversed-phase HPLC.

5 µm silica (Kromasil, Akzo Nobel, Sweden) with a specific surface area of 313 m^2^/g and pore diameter of 11 nm was used as a matrix for CSPs synthesis. Pure 3-glycidoxypropyltriethoxysilane (Sigma-Aldrich, Milwaukee, WI, USA) was used to activate silica with epoxy-functional groups.

An amino acid standard kit was purchased from Sigma-Aldrich (USA). Chromatographically pure methanol and acetonitriles were obtained from Panreac (Spain). Glacial acetic acid (pure for chemical analysis, Vekton, Moscow, Russia), ultrapure phosphotic acid, pure 25% ammonium hydroxide (both from Khimmed, Moscow, Russia), triethylamine (pure, Sigma-Aldrich, CIIIA), ultrapure perchloric acid (Reakhim, Moscow, Russia), and pure for analysis sodium dihydrophosphate (Sigma-Aldrich, CIIIA) were used as buffers and additives to eluents. Deionized water was purified by the Werner system (Leverkusen, Germany).

### 4.2. Synthesis of Chiral Phases

10 g of gel was suspended in 50 mL of 0.1 M sodium acetate buffer with pH 5.5 adjusted by adding glacial acetic acid. Then 7.8 mL of 3-glycidoxypropyltriethoxysilane was added to the suspension. The reaction mixture was vigorously stirred for 2 h and left without heating under moderate stirring for 4 days. Upon completion of the reaction, the precipitate was decanted and washed with water, ethanol, and acetone, then filtered and air-dried. The prepared epoxy-activated silica contained 7.1% carbon according to elemental analysis performed with a Vario III EL instrument (Elementar, Germany).

Then, one gram of eremomycin or its derivatives was dissolved in 50 mL of distilled water, and the pH of the solution was adjusted to 8.50 by adding 1 M potassium hydroxide (pure, Merck, Germany). This solution was added to 5 g of epoxy-activated silica and carefully mixed. The suspension was kept at room temperature for one week. After that, the sorbent was washed with water and acetone. It was then dried to constant weight in an oven at 50 °C. The data of elemental analysis for the obtained CSPs are presented in Table 1.

### 4.3. Chromatographic Experiments

The prepared adsorbents were slurry packed in 250 × 4.0 mm ID stainless steel columns at a constant pressure of 60 MPa using an air-driven liquid high-pressure pump (Haskel, Burbank, CA, USA) with methanol used as a solvent. The HPLC system consisted of a Smartline 1000 gradient pump, Smartline 2500 UV-Vis detector, and injection valve equipped with a 10 µL sample loop (all from Knauer GmbH, Berlin, Germany) was used. All solvents were of chromatographic grade purity, including acetonitrile, methanol, isopropanol, *n*-propanol, and ethanol (AppliChem, Germany). Deionized water from the Milli-Q system (Millipore, Bedford, MA, USA) was used to prepare solutions and eluents.

The chemical purity and concentration of macrocyclic glycopeptide antibiotics in reaction mixtures were determined by reversed-phase HPLC using a 250 × 4.6 mm ID column packed with 5 µm Kromasil-100-C18 (Nouryon, Sweden). The column was initially equilibrated with 0.05% trifluoroacetic acid (Solvent A). A linear gradient elution from 5% to 25% of acetonitrile (Solvent B) was applied for 20 min at a flow rate of 1 mL/min at room temperature. The chromatographic peaks were detected at 280 nm.

The column void volume was calculated as an average of 5 consecutive injections of toluene solution in methanol with 100% methanol as an eluent. Void volumes of 2.214 mL, 2.228 mL, 2.267 mL, and 1.933 mL were obtained for the columns packed with E-CSP, Amide-E-CSP, Adamantylamide-E-CSP, and Chloro-E-CSP, respectively.

## Figures and Tables

**Table 1:** The elemental analysis results and corresponding concentrations of glycopeptides antibiotics and their derivatives in the prepared CSP.

Antibiotic	Adsorbent	C, %	ΔC, %	Concentration of Bonded Antibiotics	
				μmol/g	Group/nm^2^
Eremomycin	E-CSP	12.23	7.03	80.3	0.20
Eremomycin amide	Amide-E-CSP	11.65	6.45	73.6	0.18
Eremomycin adamantylamide	Adamantylamide-E-CSP	12.82	7.65	76.8	0.19
Chloreremomycin	Chloro-E-CSP	15.07	9.26	91.5	0.22

**Table 2:** Enantioselectivity (**α**) and resolution (***R_S_***) for amino acids for the studied CSPs, eluent 20% methanol—80% 0.1 M sodium dihydrophosphate buffer with pH 4.5.

Chiral Stationary Phases	E-CSP			Chloro-E-CSP			Amide-E-CSP			Adamantylamide-E-CSP		
Amino Acid	*k’_L_*	α	*R_S_*	*k’_L_*	α	*R_S_*	*k’_L_*	α	*R_S_*	*k’_L_*	α	*R_S_*
Alanine	0.23	2.70	1.50	0.37	3.48	2.81	0.13	2.85	1.17	0.15	2.36	2.04
Valine	0.22	3.33	2.02	0.34	2.87	2.32	0.12	3.12	1.45	0.15	2.75	2.02
Norvaline	0.33	2.71	1.70	0.30	2.94	2.27	0.23	2.52	1.23	0.22	2.12	1.37
Leucine	0.31	2.38	1.68	0.32	2.06	1.90	0.24	2.31	1.52	0.23	2.00	1.28
Isoleucine	0.24	3.70	2.26	0.30	3.29	3.16	0.13	3.17	1.44	0.12	2.65	1.77
Norleucine	0.34	2.05	1.31	0.30	1.83	1.85	0.23	1.81	0.69	0.24	1.64	0.86
Tryptophan	2.83	1.76	2.20	2.38	1.10	0.82	1.82	1.62	2.06	1.60	1.66	3.00
Tyrosine	0.83	4.25	4.35	0.79	1.24	0.81	0.66	3.87	4.66	0.54	3.41	0.53
Phenylalanine	0.76	3.79	2.71	0.71	1.31	0.82	0.55	3.44	3.72	0.44	2.83	0.56
Aspartic acid	2.18	1.51	1.50	2.92	1.47	1.62	1.47	1.50	1.30	1.30	1.38	1.03
Glutamic acid	1.82	2.29	2.60	2.11	2.39	3.51	1.11	2.05	2.32	1.14	2.11	2.55
Histidine	0.23	1.42	0.35	0.30	1.000	0.00	0.14	1.32	0.53	0.12	1.27	0.62
Lysine	0.0	1.0	0.0	0.30	1.000	0.0	0.0	1.0	0.0	0.0	1.0	0.4
Arginine	0.0	1.0	0.0	0.30	1.000	0.0	0.0	1.0	0.0	0.0	1.0	0.0
Ornithine	0.0	1.0	0.0	0.30	1.000	0.0	0.0	1.0	0.0	0.0	0.0	0.0
Proline	0.44	9.0	5.22	0.67	2.26	1.73	0.22	7.01	6.97	0.22	8.69	5.35
Cysteine	0.35	2.47	1.62	0.60	1.63	0.91	0.26	1.30	0.50	0.24	1.61	0.82
Asparagine	0.34	1.00	0.0	0.34	1.43	0.91	0.0	1.00	0.0	0.0	1.05	0.67
Glutamine	0.23	2.81	1.91	0.34	1.75	1.37	0.15	2.90	1.33	0.16	2.33	1.14
Serine	0.25	1.52	0.62	0.33	1.60	0.88	0.14	1.53	0.44	0.13	1.27	0.71
Threonine	0.15	1.50	0.41	0.30	1.84	1.10	0.14	1.43	0.41	0.14	1.33	0.35
Methionine	0.32	2.09	1.61	0.47	2.05	1.88	0.22	1.91	1.00	0.20	1.78	1.32

**Table 3:** Enantioselectivity and resolution for amino acids obtained with Chloro-E-CSP depending on the composition of the mobile phase (0.7 mL/min flow rate, 25 °C, *t*_0_ = 2.701).

Mobile Phase	10% MeOH^−^ 0.1 M NaH_2_PO_4_ pH 4.5		Water		10% MeOH^−^ 0.1% HClO_4_ pH 4.5 (NH_4_OH)		10% MeOH^−^ Water		10% MeOH^−^ 0.1%TFA	
Amino Acid	$k_{L}^{\prime }$	α	$k_{L}^{\prime }$	α	$k_{L}^{\prime }$	α	$k_{L}^{\prime }$	α	$k_{L}^{\prime }$	α
Leucine	0.35	2.59	0.30	2.00	0.41	3.73	0.30	2.39	0.30	2.00
Valine	0.31	4.09	0.30	3.59	0.30	3.69	0.30	3.83	0.30	1.72
Cysteine	0.31	2.00	0.41	2.08	0.41	2.25	0.41	2.14	0.30	1.00
Asparagine	0.26	1.51	0.30	1.40	0.41	1.51	0.30	1.44	0.30	1.00
Methionine	0.34	2.21	1.09	3.09	0.53	1.62	0.41	2.94	0.30	1.76
Tryptophan	2.04	1.11	3.36	1.03	3.95	1.24	3.12	1.00	0.30	1.00
Phenylalanine	0.59	1.31	0.77	1.00	0.77	1.05	0.77	1.00	0.77	1.00
Aspartic acid	2.63	1.44	-	-	3.83	1.38	-	-	0.53	1.33
Alanine	0.25	3.79	1.09	1.60	0.97	1.95	-	-	-	-
Lysine	0.09	1.74	4.71	1.00	1.00	1.00				

**Table 4:** Effects of pH on enantioselectivity, retention, and resolution for Chloro-E-CSP.

Amino Acid	10% MeOH, 0.2 M Sodium Phosphate Buffer											
	pH 3.6			pH 4.5			pH 7.2			pH 8.3		
	$k_{L}^{\prime }$	α	*R_S_*	$k_{L}^{\prime }$	α	*R_S_*	$k_{L}^{\prime }$	α	*R_S_*	$k_{L}^{\prime }$	α	*R_S_*
Leucine	0.30	2.27	2.1	0.30	2.23	2.0	0.30	2.11	2.0	0.30	1.92	1.9
Valine	0.30	3.47	2.9	0.30	3.63	3.0	0.30	3.55	2.9	0.30	3.55	2.9
Cysteine	0.41	1.91	1.7	0.53	2.13	1.9	0.30	1.60	1.7	0.30	1.60	1.5
Asparagine	0.30	1.72	1.0	0.30	1.60	1.1	0.30	1.56	1.1	0.30	1.60	1.0
Methionine	0.41	2.25	2.0	0.41	2.20	2.0	0.30	1.80	2.0	0.41	2.02	1.9
Tryptophan *	2.04	1.00	0.8	2.04	1.11	0.9	2.42	1.19	1.0	2.18	1.18	1.0
Phenylalanine *	0.59	1.16	0.8	0.59	1.31	0.8	0.89	1.32	0.9	0.77	1.23	0.8
Histidine	0.18	1.00	0.18	0.0	1.0	0.0	0.30	1.16	0.5	0.30	1.16	0.6
Lysine	0.18	1.00	0.18	0.0	1.0	0.0	0.30	1.08	0.3	0.30	1.20	0.5
Aspartic acid	1.12	1.42	1.5	1.47	1.44	1.5	0.41	1.34	1.6	0.18	1.00	-

*—data obtained with 0.1 M sodium phosphate buffer.

## Data Availability

Not applicable.

## Sample Availability

Samples of the compounds are not available from the authors.
